# Contiguous Genome Sequence of *Frankia* sp. Strain ArI3, Isolated from Root Nodules of Alnus rubra Bong

**DOI:** 10.1128/MRA.00800-21

**Published:** 2021-10-28

**Authors:** Callum J. Bell, Johnny A. Sena, Isaac S. Gifford, Alison M. Berry

**Affiliations:** a National Center for Genome Resources, Santa Fe, New Mexico, USA; b Department of Plant Sciences, University of California, Davis, California, USA; SIPBS, University of Strathclyde

## Abstract

We report the genome sequence of *Frankia* sp. strain ArI3, recovered as a single contig from one run of the Oxford Nanopore Technologies (ONT) MinION instrument. The genome has a G+C content of 72%, is 7,541,222 bp long, and contains 5,427 predicted protein-coding genes.

## ANNOUNCEMENT

We sequenced the genome of *Frankia* sp. strain ArI3, a nitrogen-fixing taxon (phylum *Actinobacteria*) isolated from the root nodules of Alnus rubra plants grown in a greenhouse at Harvard Forest (Petersham, MA, USA) (1). The root nodules were initiated using a crushed-nodule inoculum derived from field-grown nodules of *Alnus rubra*, provided by L. E. Baribo (Weyerhaeuser Co., Tacoma, WA). Despite having been isolated in 1978 (1) and widely used experimentally (2–8), ArI3 has not been sequenced to date.

ArI3 was grown in basal medium with propionate (BAP) (9) supplemented with 5 mM pyruvate and 5 mM morpholinepropanesulfonic acid (MOPS), as in reference 10. Hyphae were collected using a 14G syringe needle, centrifuged at 10,000 rpm for 5 min, resuspended, and homogenized by passage through a 21G needle. The homogenate was inoculated into fresh medium and grown for 1 week at 28°C with shaking at 50 rpm. Hyphae were collected as above, then resuspended in Tris-EDTA (TE) buffer.

Genomic DNA (gDNA) was extracted using the cetyltrimethylammonium bromide (CTAB) protocol in reference 10, purified by phenol-chloroform extraction, and stored in TE buffer at −20°C. The DNA was quantified using the Qubit 3.0 fluorometer with the double-stranded DNA (dsDNA) broad-range (BR) assay kit, cleaned and concentrated by mixing with 0.45× volume AMPure beads (Beckman Coulter Life Sciences) according to the manufacturer’s instructions, and used for library preparation according to the ONT 1D gDNA protocol (SQK-LSK109).

Sequencing was performed on a MinION device (MinKNOW version 3.3.2) connected to a MinIT processing unit. Guppy version 3.0.3 (11) was used for real-time base calling in high-accuracy mode, with a quality score cutoff of 7. We obtained 673,563 reads averaging 4,608 bp, with a maximum length of 109,884 bp.

The sequence reads in FASTQ format were assembled using Flye version 2.7.1 (12), specifying the expected genome size, and aligned back to the draft assembly using Minimap2 version 2.17 (13). After conversion to BAM format (14), the alignments were assessed for quality and read alignment rate using PycoQC version 2.5.0.21 (15). The assembly was polished using Racon version v1.4.16 (16), followed by a second polishing step using Medaka version 1.0.1 (https://nanoporetech.github.io/medaka/).

The genome was recovered as a single contig 7,541,222 bp long, with a 72% G+C content. In total, 3,104,208,283 nucleotides were sequenced, representing 411× coverage. Annotation of the corresponding RefSeq record at NCBI with the Prokaryotic Genome Annotation Pipeline (PGAP) (17) predicted 5,427 protein-coding genes. CheckM version 1.1.3 (18) indicated that 98% of the *Actinomycetales* marker genes were present, with zero contamination or strain heterogeneity. ArI3 is closely related to CpI1-P (19) (GenBank accession number GCA_001421075.1) and CpI1-S (19) (GCA_000948395.1), sharing digital DNA-DNA hybridization (dDDH) (20) values of 99.3% and 99.5%, respectively. Plasmids previously reported in strain ArI3 (21) were not detected in this study. Underrepresentation of plasmids in ONT sequencing has been noted previously (22). Unless noted otherwise, all software was run using default parameters.

### Data availability.

The raw data and assembled genome sequence are indexed under NCBI BioProject accession number PRJNA747286. The assembled genome sequence has been assigned GenBank accession number CP079862. The MinION reads have been assigned the Sequence Read Archive accession number SRR15181822.
